# Genome-wide association and genomic prediction identifies soybean cyst nematode resistance in common bean including a syntenic region to soybean *Rhg1* locus

**DOI:** 10.1038/s41438-018-0085-3

**Published:** 2019-01-01

**Authors:** Liwei Wen, Hao-Xun Chang, Patrick J. Brown, Leslie L. Domier, Glen L. Hartman

**Affiliations:** 1 Department of Crop Sciences, University of Illinois, Urbana, IL 61801 USA; 2 United States Department of Agriculture—Agricultural Research Service, Urbana, IL USA; 3 Present Address: Monsanto, St. Louis, MO 63167 USA; 4 Present Address: Department of Plant, Soil and Microbial Sciences, Michigan State University, East Lansing, MI 48824 USA; 5 Present Address: Department of Plant Sciences, University of California, Davis, CA 95616 USA

**Keywords:** Genome-wide association studies, Genetics

## Abstract

A genome-wide association study (GWAS) was applied to detect single nucleotide polymorphisms (SNPs) significantly associated with resistance to *Heterodera glycines* (HG) also known as the soybean cyst nematode (SCN) in the core collection of common bean, *Phaseolus vulgaris*. There were 84,416 SNPs identified in 363 common bean accessions. GWAS identified SNPs on chromosome (Chr) 1 that were significantly associated with resistance to HG type 2.5.7. These SNPs were in linkage disequilibrium with a gene cluster orthologous to the three genes at the *Rhg1* locus in soybean. A novel signal on Chr 7 was detected and associated with resistance to HG type 1.2.3.5.6.7. Genomic predictions (GPs) for resistance to these two SCN HG types in common bean achieved prediction accuracy of 0.52 and 0.41, respectively. Our study generated a high-quality SNP panel for 363 common bean accessions and demonstrated that both GWAS and GP were effective strategies to understand the genetic architecture of SCN resistance in common bean.

## Winning the war against worms

A broad genomic mapping effort in the common bean reveals sequence elements that may help protect this crop from plant-killing worms. The soybean cyst nematode has the potential to wreak havoc on the common bean, an important food source in many countries. Researchers led by Glen Hartman at the US Department of Agriculture conducted an extensive survey of 363 different derivatives of this crop in order to identify genetic features that might confer resistance against two different strains of this infectious worm. Their study identified regions on three different chromosomes that each appear to contribute some degree of protection against soybean cyst nematode. These offer starting points for understanding the molecular foundations of pest resistance in this crop, and could facilitate the future breeding of more robust bean strains.

## Introduction

Common bean (*Phaseolus vulgaris* L.) is one of the most important grain legumes in the human diet and a major source of protein for many people in developing countries^1^. Common bean has two geographical and genetic pools, one of which is the Mesoamerican gene-pool domesticated in Mexico and another is the Andean gene-pool domesticated in Central and South America^2,3^. Common bean and soybean (*Glycine max* (L.) Merr.) belong to the family *Fabaceae*, and encounter many of the same pathogens including soybean cyst nematode (SCN), *Heterodera glycines* (HG) Ichinohe^4^. For soybean, SCN is the most destructive pathogen with significant production losses worldwide^5^, and losses as high as 15% based on studies in the US^6^. SCN widely occurs in most soybean producing states in the US^7^. The top 10-producing states for soybean include North Dakota, Minnesota, and Michigan, and these states when combined make up 60% of the common bean production (http://www.usdrybeans.com/resources/production/production-facts/). In addition, much of the area planted to common bean overlaps with soybean production areas and they are planted in late spring or early summer, which coincides with the period when SCN eggs hatch to become infectious juveniles.

Successful infection of SCN on common beans has been reported in both greenhouse and field studies. One of the first reports showed that the kidney bean variety “Clark” was a host for SCN HG type 0 as this variety supported juvenile growth, enlargement, molting, and female reproduction similar to a susceptible soybean cultivar “Amsoy 71”^8^. Another greenhouse study evaluated 23 common bean accessions for resistance to two SCN populations and found one snap bean accession resistant while all the other accessions supported equal or greater cyst production compared to a susceptible soybean cultivar “Williams 79”^9^. In general, kidney beans were most susceptible to SCN followed by navy beans and pinto beans, and selected accessions of black beans were considered to be moderately resistant to SCN^10^. In addition, reduction in yields up to 50% has been reported in kidney beans, navy beans, and pinto beans in fields with high populations of SCN HG type 0^11^. The reduction of plant growth and seed yield in different bean classes to SCN infection under field conditions indicates a potential threat to the common bean industry and the need for SCN resistance in common bean.

The genetic architecture of SCN resistance in soybean has been intensively studied and reviewed^12^. Two resistance loci in soybean, *Rhg1* on chromosome (Chr) 18 and *Rhg4* on Chr 8, were repeatedly detected in bi-parental linkage mapping^13–15^. The *Rhg1* locus has been shown to have broad spectrum SCN resistance to all HG types to several resistance sources including Peking, PI437654 and PI88788^16,17^. *Rhg4* was confirmed as being necessary for full resistance to some populations of SCN (races 3 and 14) for Peking-derived resistance lines but not for PI88788. A number of minor resistance genes were also reported, which mediate quantitative resistance to different SCN HG types. For example, linkage mapping was used to identify additional loci for SCN resistance resulting in a novel locus on the opposite end of *Rhg1* on Chr 18^13^. In addition, a genome-wide association study (GWAS) using single nucleotide polymorphisms (SNPs) reported six previously found quantitative trait loci (QTL), including the *Rhg1* and *Rhg4*, along with eight novel QTL^18^. Another GWAS detected 19 SNPs significantly associated with SCN HG types 0 and 1.2.3.5.7 in a collection of 440 soybean landraces and elite cultivars, with the known SCN resistant loci, *Rhg1* and *Rhg4*, identified along with three novel loci^19^. While some mapping studies discovered leucine-rich repeat receptor-like kinase (LRR-RLK) genes that were associated with SCN resistance^19–21^, studies that functionally characterize SCN resistance genes to SCN at the *Rhg1* locus pointed out the resistance was conditioned by copy number variation of three genes including a gene encoding an α-SNAP protein^12^. The molecular genetics of SCN resistance in soybean has been a major research focus over the years whereas there is less known about SCN resistance in common bean.

SCN resistance in common bean may rely on mechanisms similar to those reported for soybean, but genetic mapping for SCN resistance in common bean by either bi-parental linkage mapping or GWAS is lacking. With the development of GWAS methodologies to access the associations between genotypic and phenotypic variations in a large population, the method has been applied to a number of traits in common bean. For example, GWAS was conducted to analyze bacterial blight resistance in common bean using 469 breeding lines and 132 SNPs; the study identified 12 significant SNPs that co-localized with previously reported QTL as well as two novel QTL^22^. In another study, the genetic architecture of five agronomic traits was investigated using 233 amplified fragment length polymorphisms (AFLPs), 80 simple sequence repeats (SSRs), and 105 SNPs in 66 common bean genotypes^23^. Nonetheless, there has been no genetic mapping studies conducted to understand SCN resistance in common bean.

The use of GWAS to detect genetic variants accounting for large phenotypic variation and to highlight a QTL interval using linkage disequilibrium (LD) among neighboring SNPs provides a powerful genetic tool for associating phenotypes and genotypes. However, GWAS may result in numerous significant SNPs scattered across the genome when the phenotypic variation is explained by multiple genetic variants with small effects. Genomic prediction (GP) compensates for this disadvantage of GWAS, and GP does not depend on the detection of significant QTL. Instead, GP accounts for all markers effects across the whole genome simultaneously in a prediction model and genomic estimated breeding values (GEBVs) based on the sum of all effects, which may result in a better prediction of phenotype than conventional single marker-assisted selection^24–26^. However, multiple statistical concerns occur when the number of predictor variables (number of SNPs) is much larger than the number of observations (number of plants phenotyped). First, there is no absolute solution to the coefficients when too many variables are included in a model. Second is collinearity since adjacent SNPs are usually correlated, resulting in over-fitting and instability of the prediction model^27,28^. To address these problems, numerous prediction methods, including parametric and nonparametric methods, have been proposed. The most popular parametric methods include penalized regression approach (ridge regression)^13,29–31^, least absolute shrinkage and selection operator (LASSO)^32^, elastic net, and Bayesian-based methods (Bayes A, Bayes B, Bayes Ridge Regression, and Bayesian LASSO)^33^. Nonparametric methods include random forest^34^ and Reproducing Kernel Hilbert Spaces regression^35^. Among all those models, ridge regression was reported to offer good performance in multivariate prediction problems^13,29–31^.

In our study, the core collection of common bean accessions was genotyped using genotyping-by-sequencing (GBS) and phenotyped against SCN HG types 2.5.7 and 1.2.3.5.6.7, the most common SCN HG types in the Midwest and northern soybean producing areas in the US, with the goal to understand the genetic architecture of SCN resistance and identify genetic loci that confer resistance to different HG types in common bean. In addition to SCN resistance, previously identified QTL for two agronomic traits (seed coat color and seed weight) were included to validate the reliability of our GWAS and GPs. GP was applied to estimate the GEBVs of common bean accessions for resistance to two SCN HG types, and prediction accuracies were evaluated using cross-validation. Our study represents the first GWAS and GP for SCN resistance in common bean.

## Results

### Phenotypic analyses for SCN resistance, seed coat color, and seed weight

**Fig. 1:**
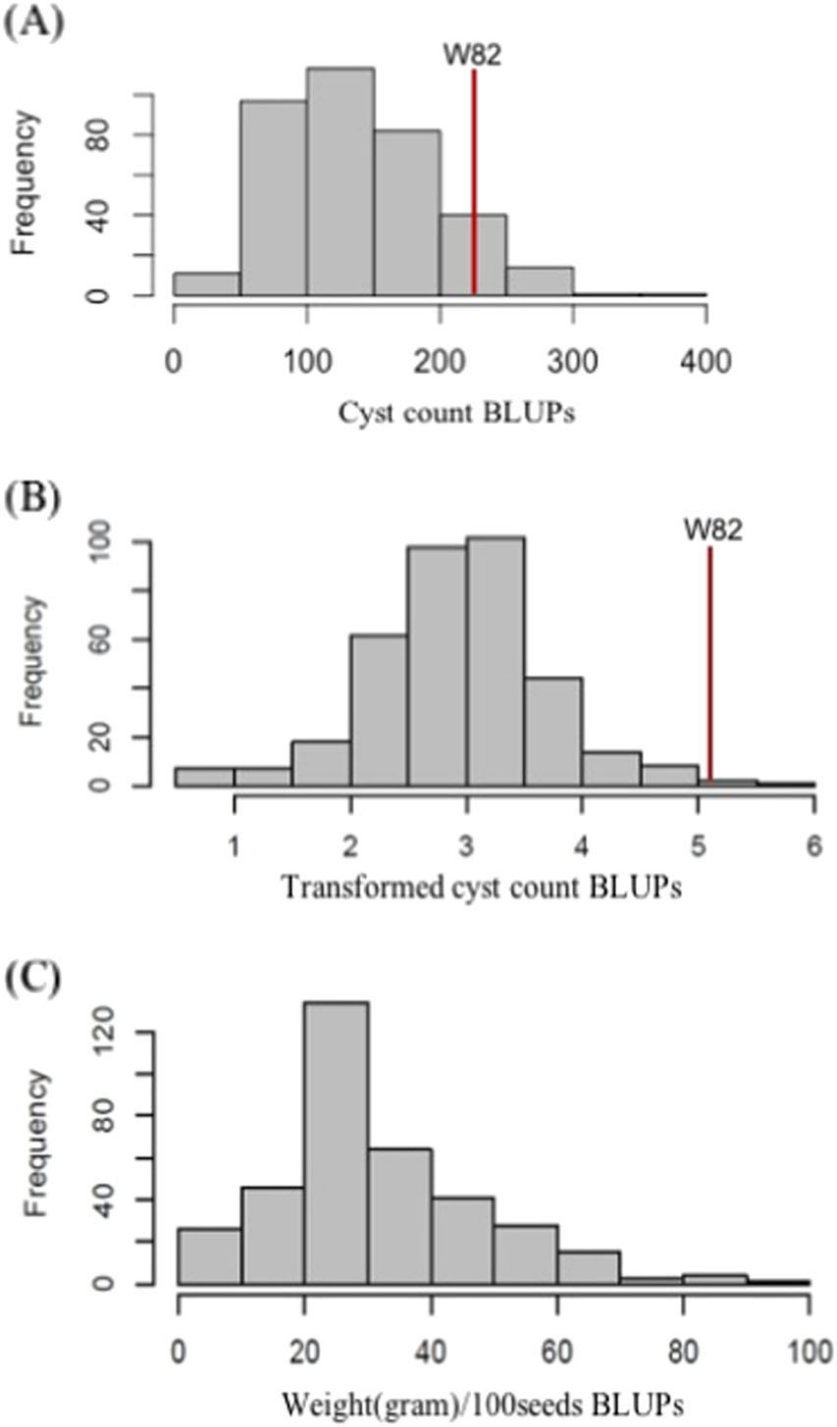
Phenotypic analyses for soybean cyst nematode (SCN) resistance, and seed weight in a panel of 363 common bean accessions. Frequency distribution of **a** HG type 2.5.7 and **b** 1.2.3.5.6.7 cyst counts best linear unbiased predictions (BLUPs). Cyst counts of the HG 1.2.3.5.6.7 were transformed to the 0.26th power before calculating BLUPs. **c** Frequency distribution of seed weight

In the phenotyping experiments for SCN resistance using the female index (FI), each accession was replicated multiple times in a random complete block design (RCBD). In order to obtain the most representative phenotypic value for each accession, a mixed model was fit to estimate a best linear unbiased prediction (BLUP), which is more accurate than the average because BLUP accounts for blocking effects across the experiments. Greenhouse evaluations of the common bean core collection for resistance to SCN HG type 2.5.7 resulted in a normal distribution with a range of BLUPs from 8 to 395 (FI from 0.5 to 198.9) (Fig. 1a). Only 16 accessions showed high resistance to SCN HG type 2.5.7 and 54 accessions showed moderate resistance. On the other hand, 160 accessions had high resistance and 164 accessions had moderate resistance to SCN HG type 1.2.3.5.6.7. The FI to SCN HG type 1.2.3.5.6.7 was left skewed and Box–Cox transformation was applied to normalize the phenotype data (Fig. 1b). The complete list of common bean accessions used and their responses to the infection of two SCN HG types are summarized in Supplementary Table 1. There were 19 accessions with white seed coats, 50 accessions with red seed coats, and 90 accessions with black seed coats. The seed weight of the 363 common bean accessions (weight of randomly selected 100 seeds) ranged from 2 to 91.6 g, with approximately a normal distribution (Fig. 1c).

### SNP calling, linkage disequilibrium (LD) decay analysis, and population structure assessment

Illumina sequencing yielded 264,276,230 raw reads, and after quality control, a total of 84,416 SNPs were obtained from SNP calling using *P. vulgaris* G19833 as the reference genome and missing SNPs were imputed using Beagle v4.1^36^. These SNPs were distributed over 11 chromosomes with an average of 7674 SNPs per chromosome (Table 1). LD decay was estimated for each chromosome and ranged from 50 to 70 kb at a cutoff of the squared correlation coefficient (*r*^2^ = 0.2) with about 10 SNPs per LD window. The high extent of LD decay in common bean was expected due to self-pollination, and is comparable to that in soybean, which was reported to be about 80 kb in wild soybeans and 130 kb in cultivated soybeans^37^.

**Table 1:** Linkage disequilibrium (LD) decay estimated for different common bean chromosomes

Chr No.^a^	Chr size (kb)	No. of SNPs^b^	LD decay (kb)^c^	SNPs per LD window^d^
1	52,183.5	8571	70	12
2	49,033.7	8559	60	10
3	52,218.6	8549	60	10
4	45,793.2	8247	65	12
5	40,237.5	7313	65	12
6	31,973.2	8600	65	17
7	51,698.4	6289	60	7
8	59,634.6	9333	50	8
9	37,399.6	5073	60	8
10	43,213.2	7662	60	11
11	50,203.6	9220	50	9

^a^Chromosome number

^b^Number of SNPs used in this study

^c^LD decay at *r*^*2*^ = 0.2

^d^Average SNPs needed was calculated by dividing chromosome size by LD decay

### Population structure

**Fig. 2:**
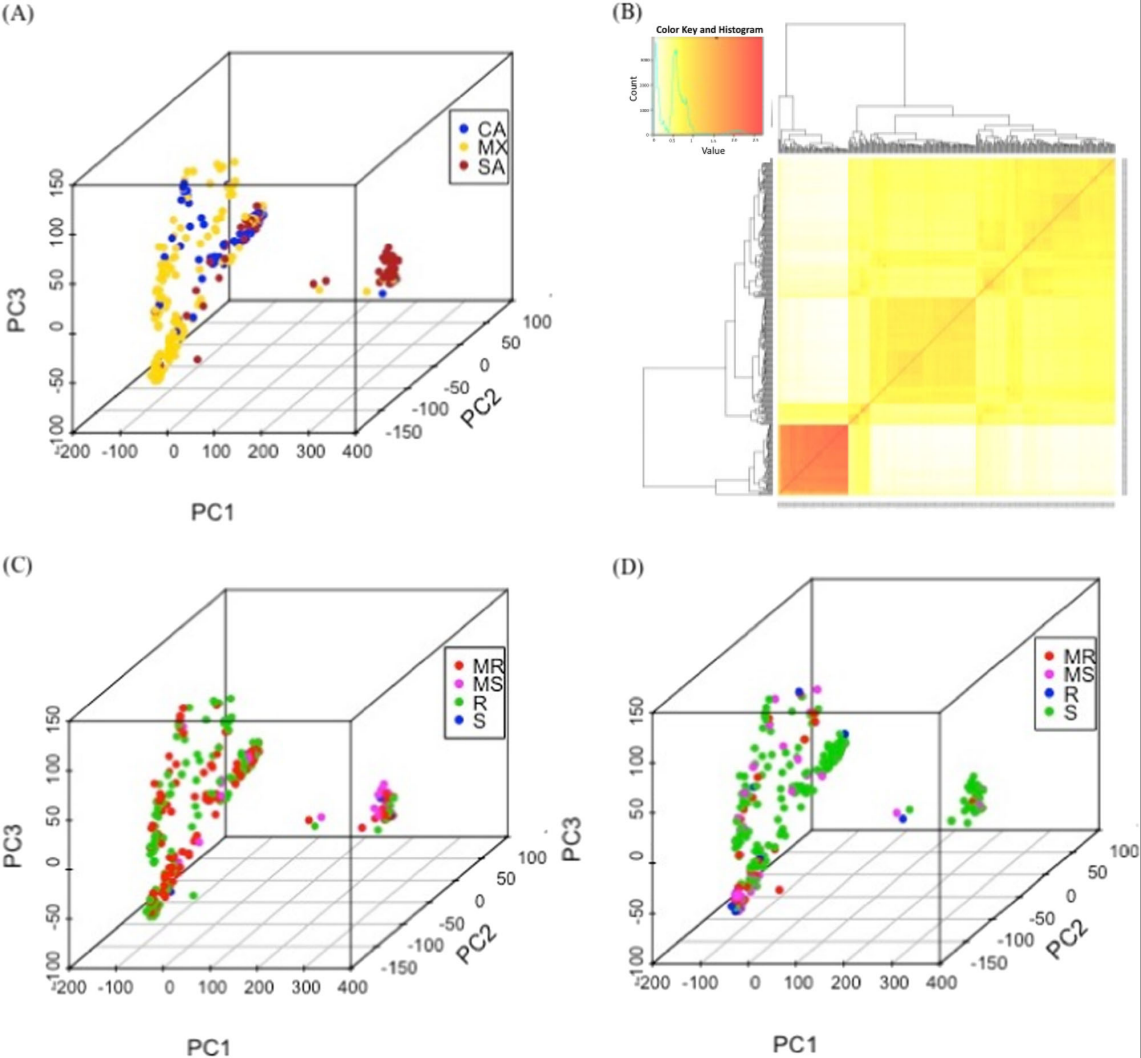
Principal component analysis and kinship matrix of the 363 common bean accessions genotyped with 84,416 single nucleotide polymorphisms. **a** Genetic variation explained by the first three principal components. Different colors represent different origins (CA: Central America; MX: Mexico; SA: Southern America), and the principal components indicate distinct population structure. **b** Kinship matrix for the 363 common bean accessions. **c** Different colors represent different levels of resistance (MR: moderately resistant; MS: moderately susceptible; R: resistant; S: susceptible) to soybean cyst nematode (SCN) HG type 2.5.7 and **d** HG type 1.2.3.5.6.7. The results showed minor confounding effect between population structure and SCN resistance

The population structure of the 363 common bean accessions was estimated by PCA using the 84,416 SNPs. Distinct subpopulations matching geographic origins were detected (Fig. 2a). The Mexico group and the Central American group had some overlap. However, the South American group clustered distinct from the other two groups. Kinship analysis with genetic relatedness among the 363 common bean accessions identified two clades, which is also consistent with the prior knowledge of two genetic pools (Fig. 2b). On the other hand, accessions with different SCN resistance levels did not cluster into these distinct subgroups, indicating a mild confounding concern between subpopulations based on geographic origins and SCN resistance (Fig. 2c, d). Bayesian information criterion (BIC)-based model selection also suggested that no principal component was required to control for population structure (Supplementary Table 2). Therefore, a unified mixed linear model (MLM) with a kinship matrix but no principal component was used for GWAS.

### GWAS for seed coat color and seed weight

**Fig. 3:**
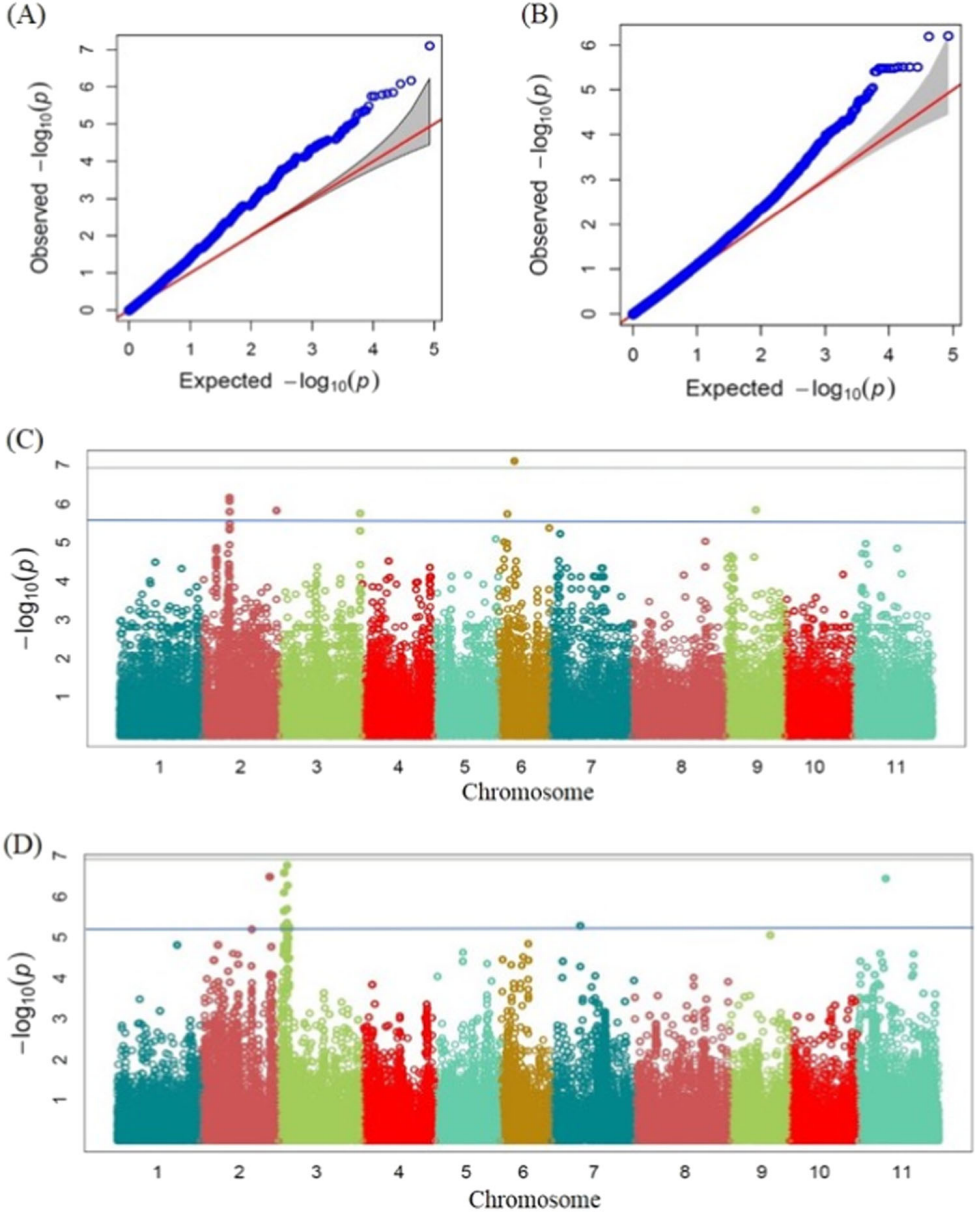
Genome-wide association study for seed coat color and seed weight. **a** Quantile–quantile (QQ) plot for seed coat color. **b** QQ plot for seed weight. **c** Manhattan plot for seed coat color. **d** Manhattan plot for seed weight. The grey horizontal line in both Manhattan plots represents the Bonferroni correction threshold, and the blue line indicates a single nucleotide polymorphism below a 5% false discovery rate adjusted *P* value

The GWAS for coat color and seed weight were compared to the results in the literature in order to validate our methodology. For seed coat color, the known locus *V* was mapped on linkage group 6 by several independent studies^38–40^. A random amplification of polymorphic DNA (RAPD) marker OD12800 on Chr 6 (marker sequence locates on 10,480,539–10,480,584 bp) linked in coupling phase with the *V* locus was reported^39^, and this RAPD marker was in the LD region with a highly significant SNP detected in our study around 9.6 Mb on Chr 6 (Table 2; Fig. 3a, b). For seed weight, GWAS identified 14 SNPs distributed over six regions on Chrs 2, 3, 7, and 11 with a FDR lower than 0.05 (Table 2; Fig. 3c, d). Previous linkage mapping studies for seed weight discovered QTL on Chrs 2, 3, 6, 7, 8, and 11 using breeding populations^41–43^. The three significant SNPs on Chr 2 (43,368,553, 43,379,956, and 43,400,258 bp) detected in our study were in LD with the candidate gene Phvul.002G282200 which was reported to be on Chr 2 between 44,603,605 and 44,608,648 bp^44^. The significant SNP on Chr 3 at 5,204,703 bp in our study was in LD with the candidate gene Phvul.003G041200 which was on Chr 3 between 4,582,905 and 4,584,971 bp^44^. The discovery of SNPs that match to previously described QTL for seed coat color and seed weight indicated the correctness of our GWAS results.

**Table 2:** Single nucleotide polymorphisms (SNPs) significantly associated with soybean cyst nematode resistance (HG type 2.5.7 and HG type 1.2.3.5.6.7.), seed coat color, and seed weight

Trait	Chr	SNP position	*P* value	Minor allele frequency	*R*^2^ of model without SNP	*R*^2^ of model with SNP	FDR-adjusted *P* value	Allele effect estimate
HG 2.5.7 SCN resistance	1	18,388,403	1.02 × 10^−6^	0.070	0.115	0.176	0.030	47.72
	1	18,388,408	1.12 × 10^−6^	0.070	0.115	0.176	0.030	47.27
	1	18,388,378	1.36 × 10^−6^	0.070	0.115	0.175	0.030	46.93
	1	18,388,392	1.53 × 10^−6^	0.068	0.115	0.174	0.030	47.02
	9	35,068,146	1.80 × 10^−6^	0.166	0.115	0.174	0.030	48.96
	1	10,061,925	4.94 × 10^−6^	0.071	0.115	0.168	0.070	−45.90
HG 1.2.3.5.6.7 SCN resistance	7	44,761,605	9.57 × 10^−7^	0.204	0.158	0.217	0.081	92.74
Seed coat color	6	9,601,167	7.95E−08	0.144	0.417	0.537	0.007	0.31
	2	17,276,368	6.89 × 10^−7^	0.403	0.417	0.519	0.017	0.23
	2	17,278,934	8.51 × 10^−7^	0.413	0.417	0.517	0.017	0.21
	9	19,120,733	1.45 × 10^−6^	0.028	0.417	0.512	0.017	−0.52
	2	47,163,511	1.53 × 10^−6^	0.106	0.417	0.512	0.017	0.62
	2	17,278,931	1.64 × 10^−6^	0.459	0.417	0.511	0.017	−0.20
	3	50,861,768	1.81 × 10^−6^	0.163	0.417	0.51	0.017	−0.84
	3	50,928,462	1.81 × 10^−6^	0.163	0.417	0.51	0.017	−0.84
	6	5,131,072	1.82 × 10^−6^	0.234	0.417	0.51	0.017	−0.49
	2	17,294,139	3.25 × 10^−6^	0.406	0.417	0.505	0.028	0.21
Seed weight^a^	3	5,204,703	6.24 × 10^−7^	0.167	0.617	0.645	0.023	7.56
	11	16,852,607	6.40 × 10^−7^	0.065	0.617	0.645	0.023	7.02
	2	43,368,553	3.09 × 10^−6^	0.193	0.617	0.641	0.023	11.92
	2	43,379,956	3.09 × 10^−6^	0.193	0.617	0.641	0.023	11.92
	2	43,400,258	3.09 × 10^−6^	0.193	0.617	0.641	0.023	11.92
	7	17,891,385	3.10 × 10^−6^	0.186	0.617	0.641	0.023	−5.54
	3	3,306,736	3.30 × 10^−6^	0.198	0.617	0.641	0.023	15.77
	3	3,306,784	3.30 × 10^−6^	0.198	0.617	0.641	0.023	15.77
	3	3,307,933	3.30 × 10^−6^	0.198	0.617	0.641	0.023	15.77
	3	3,307,946	3.30 × 10^−6^	0.198	0.617	0.641	0.023	15.77
	3	3,307,948	3.30 × 10^−6^	0.198	0.617	0.641	0.023	15.77
	3	3,335,111	3.30 × 10^−6^	0.198	0.617	0.641	0.023	15.77
	2	32,146,945	3.91 × 10^−6^	0.193	0.617	0.641	0.024	14.21
	2	32,146,953	3.91 × 10^−6^	0.193	0.617	0.641	0.024	14.21

^a^Top 15 SNPs shown

### GWAS for SCN resistance

**Fig. 4:**
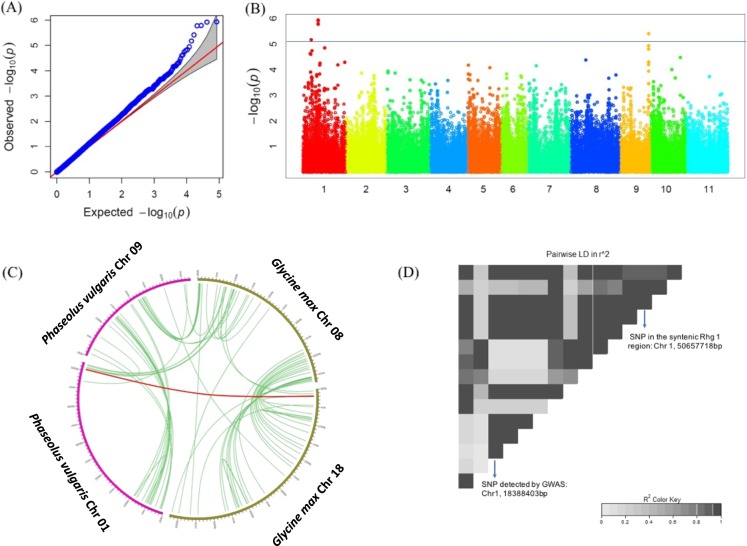
Genome-wide association study (GWAS) for soybean cyst nematode (SCN) resistance to HG type 2.5.7. **a** Quantile–quantile (QQ) plot for SCN resistance to HG type 2.5.7. **b** Manhattan plot identified multiple significant SNPs on Chr 1 and Chr 9. The blue line indicates an SNP below a 5% false discovery rate (FDR)-adjusted *P* value. **c** Synteny analysis between soybean (*Glycine max*) Chr 8 (which harbors *Rhg4*) and Chr 18 (harbors *Rhg1*) and common bean (*Phaseolus vulgaris*) Chr 1 and Chr 9. The red line indicates the soybean *Rhg1* locus at the beginning of the physical map of Chr 18 shares synteny to the bottom of common bean Chr 1. **d** Pairwise linkage disequilibrium (LD) displays of 15 single nucleotide polymorphisms (SNPs) located in the region surrounding SNPs on chromosome 1 detected by GWAS. The plot showed that the SNPs are in LD with the putative *Rhg1* gene

For SCN HG type 2.5.7, a genomic region on Chr 1 contained four SNPs with a false discovery rate (FDR) below 0.05. Using a less stringent FDR cutoff at 0.1, SNPs located at two other regions (Chr 1 and Chr 9) were found (Table 2; Fig. 4a, b). The significant SNPs on Chr 1 explained 5.9–6.1% of phenotypic variation, and additional 5.9% and 5.3% of phenotypic variation were explained by the two SNPs on another location of Chr 1 and on Chr 9, respectively (Table 2). In a comparative genomic study, three random genomic clones (Bng122, Bng126, and Bng225) located on the Chr 1 of common bean were tested as RFLP probes in soybean and these probes were mapped to the region near the *Rhg1* locus in soybean^45^. Another comparative mapping indicated linkage group D1 (Chr 1) of common bean^46^ were collinear with the top of linkage group G (Chr 18) of soybean^40^. These results indicated the genomic region on Chr 1 of common bean is a syntenic region of Chr 18 of soybean, where orthologous SCN resistance genes to soybean *Rhg1* may be found.

To further confirm our results, the sequences of the three soybean SCN resistance genes in the *Rhg1* locus were compared to the common bean genome and we found the beginning of soybean Chr 18 (the region of the *Rhg1* locus), was syntenic to the end of common bean Chr 1 using LegumeIP for syntenic analysis (Fig. 4c)^47^. Additionally, the most significant BLAST hits for the three genes in soybean *Rhg1* locus were found in a region from 50,629,261 to 50,655,828 bp on Chr 1 of common bean (Table 3). The *Rhg1* locus in soybean comprised these three genes: Glyma18g02580 (amino acid transporter), Glyma18g02590 (α-SNAP protein), and Glyma18g02610 (wound-inducible protein 12)^14^. The hit for Glyma18g02580 was a hypothetical protein PHAVU_001G248000g on Chr 1 between 50,653,407 and 50,655,828 bp. The hit for Glyma18g02590 was the hypothetical protein PHAVU_001G247900g on Chr 1 between 50,646,068 and 50,650,097 bp. The hit for Glyma18g02610 was the hypothetical protein PHAVU_001G247700g on Chr 1 between 50,629,261 and 50,630,123 bp. Interestingly, the position of the three genes in the common bean genome were inverted from those in the soybean genome. Moreover, the SNPs detected by GWAS was in high LD with the SNPs around the syntenic *Rhg1* region (Fig. 4d).

**Table 3:** Synteny between the soybean cyst nematode (SCN) resistance gene *Rhg1* on soybean chromosome 18 and the SCN resistant region on common bean chromosome 1 detected by genome-wide association study

Soybean chromosome 18			Common bean chromosome 1			Percent homology^b^	*E* value
*Rhg1* region genes	Position^a^	Annotation	Synthetic genes	Position^a^	Annotation		
Glyma18g02580	1,635,971–1,639,179	Amino acid transporter	PHAVU_001G248000g	50,653,407–50,655,828	Amino acid transporter	91%	0
Glyma18g02590	1,640,573–1,645,288	α-SNAP protein	PHAVU_001G247900g	50,646,068–50,650,097	α-SNAP protein	94%	0
Glyma18g02610	1,652,252–1,653,509	Wound-inducible protein 12	PHAVU_001G247700g	50,629,261–50,630,123	Wound-inducible protein 12	88%	9e−66

^a^Number of base pairs away from the beginning of a chromosome based on a physical map

^b^Percentage of identical amino acids between two syntenic genes

**Fig. 5:**
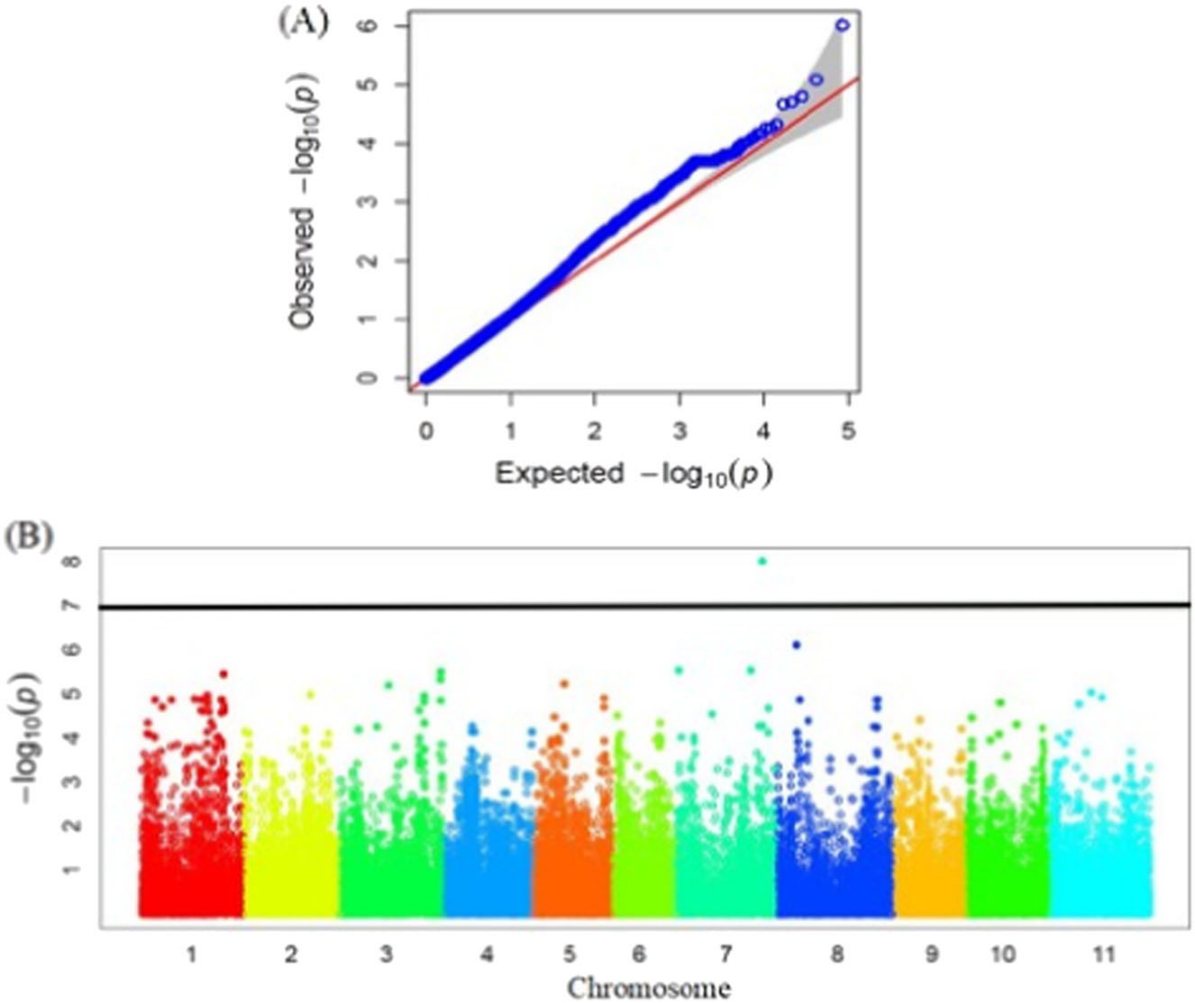
Genome-wide association study for soybean cyst nematode (SCN) resistance to HG type 1.2.3.5.6.7. **a** Quantile–quantile plot for SCN resistance to HG type 1.2.3.5.6.7. **b** Manhattan plot identified one significant single nucleotide polymorphism (SNP) on Chr 7. The black line indicates an SNP below a 10% false discovery rate adjusted *P* value

For SCN HG type 1.2.3.5.6.7, only one SNP on Chr 7 was detected below FDR at 0.1 (Fig. 5a, b). This SNP explained 5.9% of the phenotypic variation. The predicted amino acid sequences of genes at the *Rhg1* and *Rhg4* loci in soybean did not show significant similarity with the products of predicted genes proximal to the mapped SNP Chr 7 of common bean. This SNP might be a novel locus, but further studies are needed to rule out the possibility of a false-positive detection.

### GP for SCN resistance, seed coat color, and seed weight

**Fig. 6:**
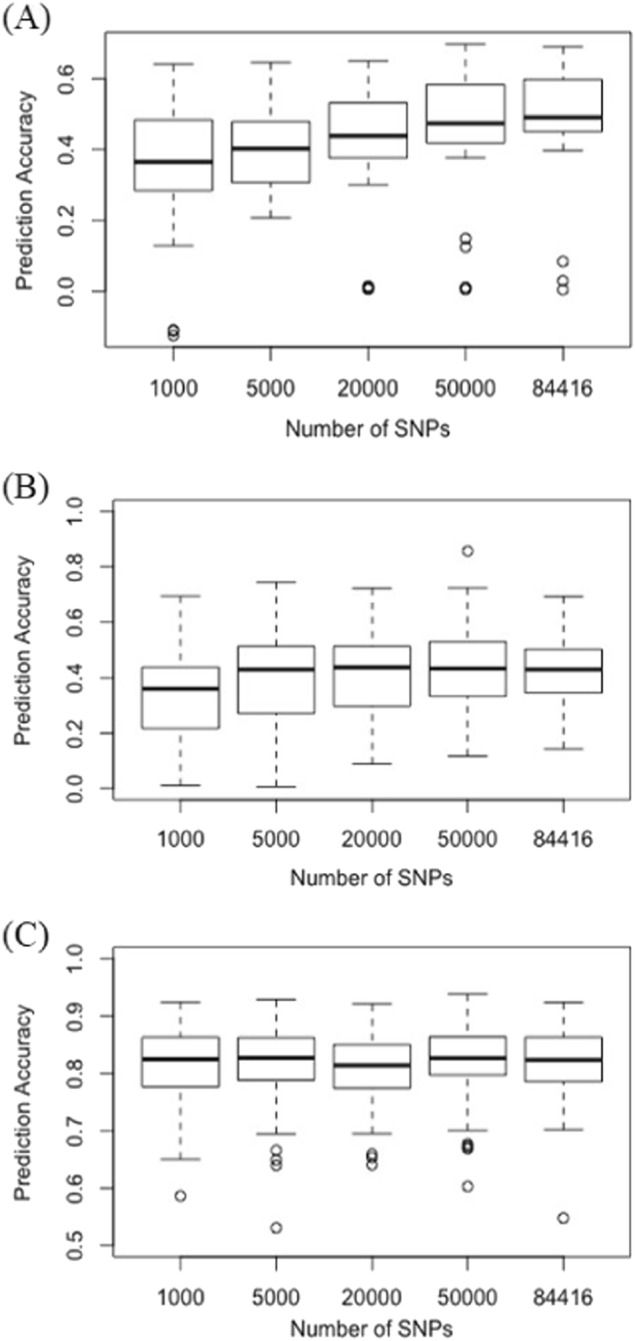
Effect of the marker density on the prediction accuracy of soybean cyst nematode. **a** HG type 2.5.7., **b** HG 1.2.3.5.6.7., and **c** seed weight in common bean. For resistance to HG type 2.5.7., prediction accuracy slightly decreased when the number of markers was reduced to 5000 and 1000. For resistance to HG type 1.2.3.5.6.7., only the prediction accuracy with 1000 single nucleotide polymorphisms showed decreased prediction accuracy. Prediction accuracy for seed weight was not affected by number of single SNPs

Besides identifying SNPs associated with SCN resistance, seed coat color, and seed weight using GWAS, the effect of all the SNP markers were evaluated by GP models to predict the two quantitative traits: SCN resistance and seed weight. The average prediction accuracy of the models estimated by cross-validation was 0.52, 0.41, and 0.82 for SCN HG type 2.5.7, HG type 1.2.3.5.6.7, and seed weight (Supplemental Tables 3–5, respectively). The prediction accuracies were not significantly affected by the number of SNP markers. For resistance to HG type 2.5.7, a slight decrease in prediction accuracy was observed when number of markers were reduced to 5000 and 1000 (Fig. 6a). For resistance to HG type 1.2.3.5.6.7, only the prediction accuracy with 1000 SNPs showed decreased prediction accuracy (Fig. 6b), which indicated possible redundancy of the markers due to high LD. While the prediction accuracy for seed weight was not affected by number of SNPs (Fig. 6c), indicating seed weight is a quantitative trait controlled by many small effect variances.

## Discussion

The identification of SNPs associated with SCN resistance can not only help in the understanding of genetic architecture in common bean, but also facilitates the genetic improvement of cultivars and the identification of resistance. In this study, 363 common bean accessions in the USDA core collection were evaluated for their responses to two SCN HG types as well as two agronomic traits, seed coat color and seed weight. We report SNPs associated with SCN resistance to two SCN HG types using GWAS. The significant SNPs identified for resistance to HG type 2.5.7 were in LD with a cluster of genes syntenic to the *Rhg1* locus in soybean^45,48^. The genomic region in common bean was conserved with the genomic region near the SCN resistance locus *Rhg1*, and the homologous genes in common bean were inversely positioned compared to the three genes at the *Rhg1* locus of soybean. It was proposed that soybean underwent a major genome duplication about 11 million years ago after it diverged from a common bean ancestor^49^. Comparative genomics studies reported 55 syntenic blocks between the two species^2^. It was shown that the linkage group D1 (Chr 1) of common bean was collinear with the top of linkage group G (Chr 18) of soybean^45^, which is consistent with our synteny analysis. Our finding suggested a gene cluster in Chr 1 of common bean that governs SCN resistance is syntenic to the *Rhg1* locus in soybean. The study did not identify syntenic regions to the *Rhg4* locus in common bean, and there are several possible reasons for this. Population size affects the power of GWAS especially when the effect or contribution of orthologous or syntenic *Rhg4* gene is smaller than *Rhg1*. Alternatively, if the minor allele frequency of orthologous or syntenic *Rhg4* gene is small, a bigger population would be needed. It is also possible that the *Rhg4* gene exists in the common legume ancestor but lost in common bean during evolution or in the process of domestication. Future studies may focus on searching additional SCN resistance sources in common bean including for those orthologous to *Rhg4*.

The prediction accuracy of GP for seed weight was as high as 82%. The estimation of the prediction accuracies for resistance to SCN HG type 2.5.7 and HG type 1.2.3.5.6.7 were 52% and 41%, respectively, which was lower than the prediction accuracies for SCN resistance in soybean that ranged from 59 to 67%^13^. The prediction accuracies of the two agronomic traits confirmed that traits with high heritability would have higher prediction accuracy^50^. Our study provided GP on disease resistance and agronomic traits in common bean and shows how GP would a useful tool for common bean breeding programs especially for traits with high heritability.

We acquired high-density and high-quality SNPs for the 363 common bean accessions using GBS, and identified SNPs associated with resistance to two SCN HG types. Our results detected the SCN resistance for two HG types located on different locations of the Chr 1, 7, and 9. The results of our study provided the first insight into the genetic architecture of SCN resistance in common bean, and we are also the first to demonstrate the merit of applying GP to predict SCN resistance and seed weight for 363 common bean accessions. The use of GP for other quantitative traits should be useful in assisting selection and accelerating breeding in common bean.

## Materials and methods

### Plant materials and DNA preparation

A total of 363 common bean accessions representing the Mesoamerican and the Andean gene pools were included in this study. The plant panel contained a total of 171 accessions of the Central/South American core collection and 191 accessions of the Mexico core collection^51,52^ that were obtained from the USDA/ARS Western Regional Plant Introduction Station (Pullman, WA, USA). The accession G19833 from which the common bean reference genome sequence was determined^44^ was obtained from the International Center for Tropical Agriculture, Cali, Colombia.

Two seeds of each common bean accession were germinated and grown in dark to reduce chlorophyll production. Emerging trifoliate leaves were collected 5 days after planting and immediately lyophilized. Genomic DNA was extracted from freeze-dried leaf tissue using a standard CTAB protocol^53^. Genomic DNA was quantified in 96-well plates using PicoGreen (Invitrogen, Carlsbad, CA) and was normalized to 20 ng/μl. A total of 500 ng DNA of each accession in a 96-well plate was digested by *Hin*dIII and *Bfa*I restriction enzymes (New England Biolabs, Ipswich, MA), and 0.1 μM A1 adapter and 10 μM A2 adapters^54^ were used for ligation in each well. Genomic libraries were pooled and cleaned up using a QIAquick PCR purification kit (Qiagen, Valencia, CA), followed by an amplification step for 12 cycles using Phusion DNA polymerase (New England Biolabs). Average fragment size was estimated on a Bioanalyzer 2100 (Agilent, Santa Clara, CA) using a DNA1000 chip followed by a second column-cleaning.

### Genotyping-by-sequencing (GBS)

Pooled libraries were adjusted to 10 nmol and sequenced with 100-bp single-end reads in one lane of HiSeq2500 (Illumina, San Diego, CA). SNP calling was performed using Tassel5 GBS v2 variant calling pipeline IGST-GBS^55,56^. All reads were trimmed to 64 nt at the 3′ end to make sure each base has Phred score greater than 30, and the trimmed sequence were aligned to the non-masked reference genome of *P. vulgaris* G19833 *Pvulgaris* v1.0 obtained from Phytozome v11.0^44,55^ using bowtie2 with the very-sensitive mode, which is computationally slower but more sensitive and more accurate than the default sensitive mode^57^. Missing SNPs were impute using BEAGLE version 4.1^58^. Insertion–deletion polymorphisms (Indels), SNPs with minor allele frequency (MAF) less than 0.05, and SNPs with heterozygosity greater than 0.05 were excluded from GWAS and GP analyses.

### Phenotyping for SCN resistance

The 363 common bean accessions along with a soybean cultivar “Williams 82” were planted in polyvinyl chloride tubes (3 cm diameter × 15 cm deep) and 18–19 tubes were randomly inserted in a plastic container (20 cm diameter × 25 cm deep) filled with pasteurized torpedo sand. Tubes without germination were replaced with extra seedlings from containers. Each tube is an experiment unit, and each plant at 1-week-old stage was inoculated with 1 ml suspension containing approximately 2000 eggs of one SCN HG type (HG 2.5.7 or HG 1.2.3.5.6.7). All plants were maintained in 28 °C water baths with 16-h light in the greenhouse. Thirty-five days after inoculation, roots were washed, and cysts were collected from each plant. Cysts were counted under a dissecting microscope (Olympus SZX16), and the number of cysts on each plant was recorded. FI was calculated by dividing the mean number of females that developed on a tested accession by the mean number of females on the susceptible check “Williams 82”, multiplied by 100. High SCN resistance is determined at FI below 10, and moderate SCN is determined at FI between 10 and 30^59,60^. The Box–Cox method was performed to transform non-normally distributed traits such as SCN HG type 1.2.3.5.6.7 resistance, and then a mixed model was fit to estimate the BLUP for each trait.

### Phenotyping for two agronomic traits

There were two replications in this experiment, and replication was achieved over time. Because of the complexity of seed coat color in common bean^44^, only black, red, and white seeds were included in GWAS, with black seeds assigned as 2, red seeds assigned as 1, and white seeds assigned as 0. Seed weight data were obtained from the Germplasm Resources Information Network (GRIN) (www.ars-grin.gov), and the seed weight of each accession was represented by the weight of 100 randomly selected seeds of that accession.

### Genome-wide association study and genomic prediction

GWAS was performed using the R package “Genomic association and prediction integrated tool version 2 (GAPIT2)”^61^. Principal component analysis (PCA) was assessed to control potential population structure, and a kinship matrix was calculated to determine relatedness among individuals^62^. A unified MLM was used that included both kinship and PCA. The BIC was calculated in GAPIT to determine the number of principal components that should be included in the model. All SNPs with FDR below 0.1 were reported. The R package “Ridge-regression best linear unbiased prediction (rrBLUP)” was applied to estimate SNP effects by solving the MLM through the REML method (R Development Core Team 2005). The GP model was trained using ten-fold cross-validation on a training dataset, and the performance of the GP model was estimated by validating the trained prediction model on a testing dataset. Ten percent of the 363 accessions were randomly selected as the testing dataset, and they were set aside and not used for model training. The rest of the accessions were split into ten similar-sized subsets for ten-fold cross-validation. In each model training process, the SNP effects were estimated for predicting the GEBVs of accessions in the validation dataset. The ten-fold cross-validation process was then repeated for 100 iterations, and the predicted GEBVs were averaged over the 100 iterations. Prediction accuracy was calculated as the correlation between GEBVs and true phenotypic values. The effect of SNP number on prediction accuracy was estimated by including different numbers of SNPs (1000, 5000, 20,000, 50,000, and 84,416 SNPs) for GP, and a similar number of SNPs were randomly selected from each Chr.

## Electronic supplementary material

Supplementary Table 1: Complete list of common bean accessions used for genome wide association analysis (GWAS) and their response to two HG types of soybean cyst nematode (SCN) infection as of female

Supplementary Table 2. Bayesian information criterion (BIC)-based model selection for deciding the optimal number of PCs in the final model. Largest BIC value indicates best model

Supplementary Table 3 Actual cyst count and predicted cyst count of HG type 2.5.7 by genomic prediction model on common bean accessions in the testing data set

Supplementary Table 4 Actual cyst count and predicted cyst count of HG type 1.2.3.5.6.7 by genomic prediction model on common bean accessions in the testing data set

Supplementary Table 5 Actual weight (gram) per 100 seeds and predicted weight (gram) per 100 seeds by genomic prediction model of the common bean accessions in the testing data set

Dear Editor

## References

[1] Broughton WJ (2003). Beans (*Phaseolus* spp.)—model food legumes. Plant Soil.

[2] McClean, P. E., Kami, J. & Gepts, P. Genomics and genetic diversity in common bean. In *Legume Crop Genomics* (eds Wilson, R. et al.) 60–82 (AOCS Press, Champaign, IL, USA, 2004).

[3] Singh SP, Gepts P, Debouck DG (1991). Races of common bean (*Phaseolus vulgaris*, Fabaceae). Econ. Bot..

[4] Singh SP, Schwartz HF (2010). Breeding common bean for resistance to diseases: a review. Crop Sci..

[5] Hartman, G. L. Worldwide importance of soybean pathogens and pests. In *Compendium of Soybean Diseases and Pests* (eds Hartman, G. L. et al.) 4–5 (American Phytopathological Society, St. Paul, 2015).

[6] McCarville MT, Marett C, Mullaney M, Gebhart G, Tylka GL (2017). Increase in soybean cyst nematode virulence and reproduction on resistant soybean varieties in Iowa from 2001 to 2015 and the effects on soybean yields. Plant Health Prog..

[7] Tylka GL, Marett C (2017). Known distribution of the soybean cyst nematode, *Heterodera glycines*, in the United States and Canada, 1954 to 2017. Plant Health Prog..

[8] Abawi GS, Jacobsen BJ (1984). Effect of initial inoculum densities of *Heterodera glycines* on growth of soybean and kidney bean and their efficiency as hosts under greenhouse conditions. Phytopathology.

[9] Melton TA, Noel GR, Jacobsen BJ, Hagedorn DJ (1985). Comparative host suitabilities of snap beans to the soybean cyst nematode (*Heterodera glycines*). Plant Dis..

[10] Poromarto S, Nelson B (2009). Reproduction of soybean cyst nematode on dry bean cultivars adapted to North Dakota and northern Minnesota. Plant Dis..

[11] Poromarto S, Nelson B, Goswami R (2010). Effect of soybean cyst nematode on growth of dry bean in the field. Plant Dis..

[12] Mitchum MG (2016). Soybean resistance to the soybean cytst nematode *Heterodera glycines*: an update. Phytopathology.

[13] Bao Y (2014). Potential of association mapping and genomic selection to explore PI 88788 derived soybean cyst nematode resistance. Plant Genome.

[14] Cook DE (2012). Copy number variation of multiple genes at Rhg1 mediates nematode resistance in soybean. Science.

[15] Liu S (2012). A soybean cyst nematode resistance gene points to a new mechanism of plant resistance to pathogens. Nature.

[16] Concibido VC, Diers BW, Arelli PR (2004). A decade of QTL mapping for cyst nematode resistance in soybean. Crop Sci..

[17] Guo B, Sleper D, Arelli PR, Shannon JG, Nguyen HT (2005). Identification of QTLs associated with resistance to soybean cyst nematode races 2, 3 and 5 in soybean PI 90763. Theor. Appl. Genet..

[18] Vuong TD (2015). Genetic architecture of cyst nematode resistance revealed by genome-wide association study in soybean. BMC Genom..

[19] Han Y (2015). Genetic characteristics of soybean resistance to HG type 0 and HG type 1.2.3.5.7 of the cyst nematode analyzed by genome-wide association mapping. BMC Genom..

[20] Chang HX, Lipka A, Domier LL, Hartman GL (2016). Characterization of disease resistance loci in the USDA Soybean Germplasm collection using genome-wide associations. Phytopathology.

[21] Zhang H, Song BH (2017). RNA-seq data comparisons of wild soybean genotypes in response to soybean cyst nematode (*Heterodera glycines*). Genom. Data.

[22] Shi C, Navabi A, Yu K (2011). Association mapping of common bacterial blight resistance QTL in Ontario bean breeding populations. BMC Plant Biol..

[23] Nemli S (2014). Association mapping for five agronomic traits in the common bean (*Phaseolus vulgaris* L.). J. Sci. Food Agr..

[24] Crossa J (2011). Genomic selection and prediction in plant breeding. J. Crop Improv..

[25] Desta ZA, Ortiz R (2014). Genomic selection: genome-wide prediction in plant improvement. Trends Plant Sci..

[26] Newell MA, Jannink JL (2014). Genomic selection in plant breeding. Methods Mol. Biol..

[27] Chapman BP, Weiss A, Duberstein P (2016). Statistical learning for high dimensional prediction: application to criterion-keyed scale development. Psychol. Methods.

[28] Gianola D (2013). Priors in whole-genome regression: the Bayesian alphabet returns. Genetics.

[29] Chang HX, Brown P, Lipka A, Domier LL, Hartman GL (2016). Genome-wide association and genomic prediction identifies associated loci and predicts the sensitivity of *Tobacco ringspot virus* in soybean plant introductions. BMC Genom..

[30] Lorenz AJ, Smith KP, Jannink JL (2012). Potential and optimization of genomic selection for Fusarium head blight resistance in six-row barley. Crop Sci..

[31] Rutkoski, J. et al. Genomic selection for quantitative adult plant stem rust resistance in wheat. *Plant Genome* **7**, https://dx.doi.org/10.3835/plantgenome2014.02.0006 (2014).

[32] Tibshirani R (1996). Regression shrinkage and selection via the Lasso. J. R. Stat. Soc. Ser. B Stat. Methodol..

[33] Yi N, Xu S (2008). Bayesian LASSO for quantitative trait loci mapping. Genetics.

[34] Svetnik V, Liaw A, Tong C, Culberson JC, Sheridan RP (2003). Random forest: a classification and regression tool for compound classification and QSAR modelling. J. Chem. Inf. Comput. Sci..

[35] De los Campos G, Gianola D, Rosa GJ, Weigel KA, Crossa J (2010). Semi-parametric genomic-enabled prediction of genetic values using reproducing kernel Hilbert spaces methods. Genet. Res..

[36] Browning SR, Browning BL (2007). Rapid and accurate haplotype phasing and missing-data inference for whole-genome association studies by use of localized haplotype clustering. Am. J. Hum. Genet..

[37] Wang J (2016). Development and application of a novel genome-wide SNP array reveals domestication history in soybean. Sci. Rep..

[38] Bassett MJ (1997). A new allele (*Vwf*) at the V locus for flower and seed coat color in common bean. J. Am. Soc. Hortic. Sci..

[39] McClean PE, Lee RK, Otto C, Gepts P, Bassett MJ (2002). Molecular and phenotypic mapping of genes controlling seed coat pattern and color in common bean (*Phaseolus vulgaris* L.). J. Hered..

[40] Nodari RO, Tsail SM, Gilbertson RL, Gepts P (1993). Towards an integrated linkage map of common bean 2. Development of an RFLP-based linkage map. Theor. Appl. Genet..

[41] Blair MW, Iriarte G, Beebe S (2006). QTL analysis of yield traits in an advanced backcross population derived from a cultivated Andean wild common bean (*Phaseolus vulgaris* L.) cross. Theor. Appl. Genet..

[42] Park SO (2000). Mapping of QTL for seed size and shape traits in common bean. J. Am. Soc. Hortic. Sci..

[43] Tsai SM (1998). QTL mapping for nodule number and common bacterial blight in *Phaseolus vulgaris* L. Plant Soil.

[44] Schmutz J (2014). A reference genome for common bean and genome-wide analysis of dual domestications. Nat. Genet..

[45] Concibido V (1995). Targeted comparative genome analysis and qualitative mapping of a major partial-resistance gene to the soybean cyst nematode. Theor. Appl. Genet..

[46] Gepts P, Bliss FA (1986). Phaseolin variability among wild and cultivated common beans (*Phaseolus vulgaris*) from Colombia. Econ. Bot..

[47] Li J, Dai X, Liu T, Zhao PX (2012). LegumeIP: an integrative database forcomparative genomics and transcriptomics of model legumes. Nucleic Acids Res..

[48] Kelly JD, Gepts P, Miklas PN, Coyne DP (2003). Tagging and mapping of genes and QTL and molecular marker-assisted selection for traits of economic importance in bean and cowpea. Field Crops Res..

[49] Schlueter JA (2004). Mining the EST databases to determine evolutionary events in the legumes and grasses. Genome.

[50] Combs E, Bernardo R (2013). Accuracy of genome wide selection for different traits with constant population size, heritability, and number of markers. Plant Gemone.

[51] Brick MA (2006). Reaction to three races of Fusarium wilt in the core collection. Crop Sci..

[52] McClean PE (2012). Population structure and genetic differentiation among the USDA common bean (*Phaseolus vulgaris* L.) core collection. Genet. Resour. Crop Evol..

[53] Doyle J, Doyle JL (1987). Genomic plant DNA preparation from fresh tissue-CTAB method. Phytochem. Bull..

[54] Thurber CS, Ma JM, Higgins RH, Brown PJ (2013). Retrospective genomic analysis of sorghum adaptation to temperate-zone grain production. Genome Biol..

[55] Glaubitz JC (2014). TASSEL-GBS: a high capacity genotyping by sequencing analysis pipeline. PLoS One.

[56] Sonah H, Bastien M, Iquira E, Tardivel A, Légaré G (2013). An improved genotyping by sequencing (GBS) approach offering increased versatility and efficiency of SNP discovery and genotyping. PLoS One.

[57] Langmead B, Salzberg SL (2012). Fast gapped-read alignment with Bowtie 2. Nat. Methods.

[58] Browning BL, Browning SR (2013). Improving the accuracy and efficiency of identity by descent detection in population data. Genetics.

[59] Niblack TL (2002). A revised classification scheme for genetically diverse populations of *Heterodera glycines*. J. Nematol..

[60] Niblack, T. L. et al. A standard greenhouse method for assessing a soybean cyst nematode resistance in soybean: SCE08 (standardized cyst evaluation 2008). In *Plant Health Progress* (Plant Management Network, 2009).

[61] Lipka A (2012). GAPIT: genome association and prediction integrated tool. Bioinformatics.

[62] Zhang Z (2010). Mixed linear model approach adapted for genome-wide association studies. Nat. Genet..

