# Supplementary material for: Genome-wide association and genomic prediction identifies soybean cyst nematode resistance in common bean including a syntenic region to soybean Rhg1 locus
Source: Hortic Res. 2019 Jan 1;6:9. doi: 10.1038/s41438-018-0085-3 (PMC6312554; doi:10.1038/s41438-018-0085-3)
Supplement: Supplementary file 1 — Supplementary Table 1: Complete list of common bean accessions used for genome wide association analysis (GWAS) and their response to two HG types of soybean cyst nematode (SCN) infection as of female [file 41438_2018_85_MOESM1_ESM.docx]

Supplementary Table 1: Complete list of common bean accessions used for genome wide association analysis (GWAS) and their response to two HG types of soybean cyst nematode (SCN) infection as of female index (FI)^a^.

| PI | FI  HG2.5.7 | FI  HG1.2.3.5.6.7 | PI | FI  HG2.5.7 | FI  HG1.2.3.5.6.7 | PI | FI  HG2.5.7 | FI  HG1.2.3.5.6.7 |
| --- | --- | --- | --- | --- | --- | --- | --- | --- |
| G19833 | 100.00 | 11.21 | PI310511 | 64.32 | 15.89 | PI319595 | 33.04 | 5.05 |
| PI150409 | 53.30 | 1.31 | PI310515 | 55.95 | 16.45 | PI319607 | 33.48 | 10.09 |
| PI150957 | 93.83 | 7.10 | PI310546 | 48.02 | 8.22 | PI319618 | 38.33 | 7.29 |
| PI151407 | 55.95 | 3.36 | PI310556 | 49.34 | 9.91 | PI319619 | 46.26 | 8.04 |
| PI152208 | 97.36 | 8.04 | PI310561 | 69.16 | 8.79 | PI319636 | 40.09 | 13.46 |
| PI152311 | 55.95 | 5.42 | PI310586 | 63.88 | 16.26 | PI319640 | 17.18 | 6.54 |
| PI165422 | 72.69 | 7.10 | PI310599 | 99.12 | 10.09 | PI319674 | 22.91 | 9.53 |
| PI165423 | 53.74 | 2.80 | PI310611 | 40.09 | 7.66 | PI319683 | 52.86 | 2.99 |
| PI165455 | 58.59 | 2.99 | PI310660 | 101.76 | 14.02 | PI319684 | 55.07 | 1.50 |
| PI165462 | 61.67 | 3.36 | PI310663 | 63.00 | 13.64 | PI325614 | 88.55 | 2.06 |
| PI165466 | 73.13 | 4.11 | PI310690 | 92.95 | 9.53 | PI325618 | 37.00 | 21.68 |
| PI181996 | 74.45 | 10.47 | PI310726 | 46.70 | 6.17 | PI325626 | 23.35 | 16.07 |
| PI182000 | 103.52 | 17.01 | PI310739 | 77.97 | 10.09 | PI325630 | 9.25 | 3.36 |
| PI182004 | 124.67 | 3.36 | PI310778 | 49.34 | 11.40 | PI325635 | 16.30 | 0.37 |
| PI189016 | 63.00 | 13.46 | PI310814 | 39.21 | 20.56 | PI325642 | 10.13 | 3.74 |
| PI189407 | 69.16 | 7.66 | PI310826 | 33.04 | 8.60 | PI325653 | 3.96 | 1.50 |
| PI189408 | 58.15 | 8.97 | PI310828 | 54.19 | 11.96 | PI325676 | 8.81 | 3.55 |
| PI190078 | 64.76 | 4.86 | PI310829 | 103.08 | 14.77 | PI325684 | 2.64 | 4.11 |
| PI194574 | 77.53 | 5.61 | PI310836 | 69.16 | 21.31 | PI325685 | 15.42 | 2.43 |
| PI195402 | 51.10 | 5.79 | PI310850 | 72.69 | 25.23 | PI325687 | 90.75 | 11.21 |
| PI195801 | 38.77 | 12.15 | PI310865 | 54.63 | 16.07 | PI325691 | 87.36 | 5.23 |
| PI196463 | 45.37 | 2.43 | PI310883 | 62.56 | 19.63 | PI325721 | 94.71 | 15.70 |
| PI197031 | 35.68 | 3.74 | PI310886 | 73.57 | 18.32 | PI325722 | 110.57 | 14.95 |
| PI198026 | 45.81 | 5.05 | PI310891 | 50.22 | 16.82 | PI325732 | 26.43 | 10.65 |
| PI198037 | 44.49 | 29.72 | PI310915 | 52.42 | 11.96 | PI325750 | 22.91 | 3.36 |
| PI200956 | 38.77 | 2.62 | PI311794 | 36.12 | 20.56 | PI326106 | 1.32 | 15.51 |
| PI200967 | 27.31 | 3.36 | PI311843 | 67.40 | 15.70 | PI326110 | 30.84 | 8.60 |
| PI201004 | 34.36 | 4.49 | PI311853 | 10.13 | 19.07 | PI345576 | 15.86 | 0.56 |
| PI201010 | 36.12 | 4.86 | PI311940 | 51.54 | 9.91 | PI345581 | 38.33 | 4.67 |
| PI201296 | 12.78 | 1.87 | PI311942 | 40.53 | 6.54 | PI346955 | 40.97 | 0.37 |
| PI201324 | 4.85 | 2.99 | PI311947 | 15.42 | 9.72 | PI346960 | 12.33 | 12.90 |
| PI201329 | 10.57 | 2.24 | PI311962 | 136.56 | 11.40 | PI355419 | 35.68 | 11.21 |
| PI201343 | 52.86 | 8.22 | PI311967 | 50.66 | 27.66 | PI387862 | 26.43 | 6.73 |
| PI201354 | 3.08 | 0.37 | PI311974 | 58.15 | 18.50 | PI387865 | 119.38 | 42.62 |
| PI201360 | 12.33 | 1.87 | PI311999 | 63.00 | 13.64 | PI399169 | 73.13 | 12.34 |
| PI201369 | 34.80 | 6.36 | PI312016 | 51.10 | 11.03 | PI406940 | 13.66 | 30.84 |
| PI201370 | 43.17 | 12.90 | PI312017 | 58.15 | 14.58 | PI415886 | 49.34 | 31.96 |
| PI201387 | 36.12 | 15.33 | PI312018 | 59.91 | 6.17 | PI415900 | 10.13 | 30.28 |
| PI201388 | 48.90 | 24.67 | PI312031 | 88.11 | 9.91 | PI415913 | 3.52 | 29.72 |
| PI201480 | 40.09 | 16.07 | PI312052 | 59.03 | 8.04 | PI415936 | 13.66 | 12.34 |
| PI202834 | 51.54 | 22.80 | PI312064 | 100.88 | 26.36 | PI415949 | 29.52 | 34.77 |
| PI202835 | 91.63 | 22.06 | PI312083 | 13.66 | 14.21 | PI415950 | 42.73 | 30.84 |
| PI203920 | 25.55 | 12.71 | PI312090 | 72.69 | 21.68 | PI415955 | 34.80 | 17.94 |
| PI203921 | 37.89 | 13.46 | PI312098 | 78.85 | 14.21 | PI415975 | 88.99 | 51.59 |
| PI203924 | 46.26 | 26.17 | PI313237 | 33.48 | 9.72 | PI415986 | 66.52 | 66.17 |
| PI203936 | 56.39 | 16.07 | PI313254 | 59.03 | 14.21 | PI415987 | 63.00 | 84.67 |
| PI203958 | 56.39 | 27.66 | PI313270 | 100.44 | 51.59 | PI416713 | 28.63 | 7.29 |
| PI206223 | 34.36 | 19.07 | PI313272 | 73.13 | 32.15 | PI417616 | 3.96 | 7.29 |
| PI207127 | 92.07 | 12.34 | PI313297 | 76.21 | 4.30 | PI417621 | 35.68 | 11.78 |
| PI207136 | 102.20 | 37.20 | PI313328 | 8.81 | 10.47 | PI417622 | 34.80 | 4.49 |
| PI207148 | 96.04 | 146.17 | PI313357 | 92.36 | 13.08 | PI417628 | 88.99 | 8.97 |
| PI207154 | 96.04 | 28.97 | PI313366 | 41.85 | 14.39 | PI417630 | 65.20 | 0.56 |
| PI207165 | 69.60 | 58.50 | PI313373 | 104.85 | 79.81 | PI417633 | 132.16 | 18.50 |
| PI207180 | 39.65 | 28.22 | PI313386 | 102.64 | 20.19 | PI417634 | 59.47 | 20.19 |
| PI207182 | 48.46 | 36.07 | PI313394 | 42.73 | 9.35 | PI417641 | 69.60 | 22.43 |
| PI207186 | 91.19 | 17.20 | PI313408 | 96.48 | 13.64 | PI417645 | 78.37 | 10.09 |
| PI207193 | 109.69 | 24.86 | PI313412 | 40.97 | 8.04 | PI417647 | 55.51 | 3.93 |
| PI207203 | 88.11 | 44.86 | PI313429 | 15.42 | 12.34 | PI417653 | 122.47 | 14.02 |
| PI207207 | 88.55 | 26.17 | PI313433 | 90.31 | 6.36 | PI417654 | 44.93 | 3.36 |
| PI207216 | 54.19 | 7.85 | PI313440 | 8.81 | 17.20 | PI417657 | 22.03 | 3.93 |
| PI207253 | 28.19 | 7.29 | PI313444 | 16.74 | 10.28 | PI417667 | 24.23 | 16.82 |
| PI207279 | 88.55 | 75.70 | PI313445 | 0.44 | 0.00 | PI417679 | 90.31 | 15.14 |
| PI207300 | 104.41 | 24.30 | PI313458 | 64.76 | 1.50 | PI417707 | 38.77 | 17.94 |
| PI207336 | 92.95 | 10.84 | PI313459 | 22.91 | 5.79 | PI417708 | 34.80 | 15.14 |
| PI207373 | 92.51 | 14.39 | PI313470 | 23.79 | 4.30 | PI417716 | 33.04 | 0.00 |
| PI207389 | 43.17 | 18.69 | PI313483 | 75.33 | 0.00 | PI417721 | 11.89 | 13.46 |
| PI207420 | 71.81 | 17.94 | PI313486 | 66.08 | 7.29 | PI417731 | 29.07 | 10.47 |
| PI207428 | 75.33 | 6.92 | PI313490 | 27.75 | 2.24 | PI417739 | 13.22 | 1.12 |
| PI207443 | 36.56 | 6.36 | PI313495 | 35.68 | 2.99 | PI417742 | 23.79 | 16.82 |
| PI208774 | 39.21 | 29.16 | PI313501 | 23.79 | 0.56 | PI417743 | 25.11 | 3.36 |
| PI209479 | 29.52 | 8.97 | PI313512 | 14.10 | 7.48 | PI417754 | 1.76 | 3.55 |
| PI209482 | 64.76 | 17.94 | PI313531 | 91.63 | 1.50 | PI417778 | 82.31 | 0.37 |
| PI209486 | 91.19 | 73.46 | PI313532 | 66.52 | 9.16 | PI417780 | 63.25 | 4.30 |
| PI209491 | 18.50 | 9.53 | PI313537 | 92.07 | 5.23 | PI417784 | 66.52 | 1.12 |
| PI209498 | 28.19 | 17.01 | PI313571 | 29.52 | 5.79 | PI417790 | 59.91 | 1.50 |
| PI224715 | 44.05 | 3.74 | PI313572 | 96.48 | 3.36 | PI430167 | 52.42 | 45.79 |
| PI224718 | 25.55 | 16.07 | PI313583 | 37.89 | 9.53 | PI430200 | 27.75 | 11.78 |
| PI224728 | 33.48 | 23.55 | PI313592 | 45.81 | 1.50 | PI430201 | 43.17 | 7.10 |
| PI241794 | 20.70 | 15.89 | PI313597 | 53.30 | 6.36 | PI430204 | 14.10 | 4.49 |
| PI260418 | 48.46 | 9.53 | PI313598 | 128.19 | 0.00 | PI430206 | 12.33 | 0.00 |
| PI263596 | 7.49 | 15.14 | PI313608 | 97.36 | 42.62 | PI430210 | 2.64 | 4.86 |
| PI268110 | 41.41 | 16.64 | PI313609 | 91.63 | 31.40 | PI449389 | 30.84 | 11.40 |
| PI269209 | 51.54 | 9.16 | PI313613 | 111.89 | 45.61 | PI449422 | 51.54 | 10.09 |
| PI269210 | 53.74 | 9.16 | PI313630 | 51.54 | 14.21 | PI451885 | 22.91 | 4.30 |
| PI282016 | 38.77 | 51.96 | PI313633 | 154.63 | 40.37 | PI451889 | 27.75 | 4.67 |
| PI288016 | 32.60 | 21.50 | PI313634 | 94.27 | 46.36 | PI451906 | 75.33 | 7.29 |
| PI290990 | 88.11 | 21.12 | PI313639 | 72.69 | 25.42 | PI451917 | 55.07 | 10.28 |
| PI290995 | 24.23 | 17.01 | PI313658 | 138.33 | 65.05 | PI451921 | 14.98 | 15.14 |
| PI293353 | 50.22 | 8.60 | PI313664 | 146.26 | 50.84 | PI476751 | 12.33 | 19.81 |
| PI293355 | 27.31 | 10.09 | PI313665 | 129.52 | 32.15 | PI510574 | 78.41 | 10.47 |
| PI297295 | 38.77 | 93.64 | PI313667 | 143.17 | 11.21 | PI511767 | 33.48 | 8.79 |
| PI299019 | 127.31 | 8.79 | PI313671 | 199.56 | 111.40 | PI512003 | 42.29 | 6.92 |
| PI304113 | 99.12 | 8.79 | PI313674 | 57.27 | 19.44 | PI533249 | 37.44 | 13.27 |
| PI307788 | 92.95 | 4.30 | PI313693 | 128.19 | 33.64 | PI533259 | 66.52 | 24.86 |
| PI307790 | 111.89 | 18.32 | PI313701 | 100.88 | 12.71 | PI533277 | 74.01 | 12.52 |
| PI307791 | 119.82 | 11.59 | PI313709 | 21.59 | 19.44 | PI533281 | 70.93 | 32.90 |
| PI307806 | 148.46 | 7.48 | PI313720 | 113.66 | 29.16 | PI533286 | 71.81 | 12.90 |
| PI307808 | 103.52 | 12.71 | PI313727 | 71.37 | 29.16 | PI533299 | 23.35 | 10.84 |
| PI307810 | 95.59 | 8.60 | PI313733 | 5.73 | 4.49 | PI533311 | 33.04 | 10.28 |
| PI307816 | 91.19 | 2.62 | PI313749 | 22.03 | 15.14 | PI533312 | 9.69 | 13.83 |
| PI307820 | 158.59 | 8.22 | PI313782 | 136.56 | 57.76 | PI533313 | 52.86 | 24.49 |
| PI307823 | 134.80 | 6.92 | PI313809 | 54.19 | 21.87 | PI533316 | 49.78 | 17.94 |
| PI308894 | 126.87 | 25.98 | PI313830 | 102.64 | 37.01 | PI533332 | 92.07 | 18.69 |
| PI308898 | 108.37 | 5.61 | PI313833 | 144.05 | 37.57 | PI533363 | 118.50 | 18.69 |
| PI308908 | 136.12 | 15.89 | PI313835 | 99.12 | 23.55 | PI533373 | 128.63 | 16.45 |
| PI309698 | 116.74 | 8.41 | PI313837 | 125.99 | 30.28 | PI533420 | 69.16 | 17.57 |
| PI309700 | 138.33 | 22.99 | PI313839 | 47.58 | 17.94 | PI533428 | 77.97 | 19.44 |
| PI309701 | 78.41 | 5.23 | PI313842 | 72.69 | 22.99 | PI533432 | 71.81 | 5.42 |
| PI309715 | 77.97 | 1.31 | PI313847 | 15.42 | 3.36 | PI533437 | 88.55 | 12.15 |
| PI309759 | 93.83 | 16.64 | PI313850 | 166.96 | 17.38 | PI533475 | 100.44 | 17.01 |
| PI309787 | 75.77 | 18.50 | PI316016 | 99.56 | 3.93 | PI533476 | 104.41 | 29.16 |
| PI309810 | 94.71 | 24.30 | PI316031 | 100.44 | 8.97 | PI533484 | 113.22 | 22.80 |
| PI309823 | 50.66 | 13.27 | PI317027 | 147.58 | 19.07 | PI533498 | 112.78 | 20.37 |
| PI309825 | 99.56 | 8.41 | PI317350 | 56.39 | 0.00 | PI533502 | 74.45 | 5.98 |
| PI309827 | 60.35 | 6.73 | PI318694 | 9.25 | 0.00 | PI533510 | 58.59 | 10.65 |
| PI309830 | 95.59 | 5.61 | PI318695 | 24.23 | 2.62 | PI533528 | 88.99 | 31.40 |
| PI309837 | 65.64 | 4.67 | PI318703 | 14.54 | 5.61 | PI533545 | 56.39 | 2.24 |
| PI309844 | 34.36 | 9.91 | PI319554 | 24.23 | 4.67 | PI533561 | 41.85 | 8.41 |
| PI309845 | 28.19 | 4.49 | PI319573 | 48.46 | 4.86 | PI533584 | 103.96 | 6.73 |
| PI309857 | 118.06 | 24.86 | PI319587 | 2.64 | 14.58 | PI535395 | 94.71 | 7.85 |
| PI309877 | 89.43 | 18.69 | PI319592 | 36.56 | 4.30 | PI557483 | 120.70 | 19.63 |

^a^ FI = (Mean number of cysts on each indicator line/mean number of cysts on Lee 74) X 100.
