# Supplementary material for: Genome-wide association and genomic prediction identifies soybean cyst nematode resistance in common bean including a syntenic region to soybean Rhg1 locus
Source: Hortic Res. 2019 Jan 1;6:9. doi: 10.1038/s41438-018-0085-3 (PMC6312554; doi:10.1038/s41438-018-0085-3)
Supplement: Supplementary file 2 — Supplementary Table 2. Bayesian information criterion (BIC)-based model selection for deciding the optimal number of PCs in the final model. Largest BIC value indicates best model [file 41438_2018_85_MOESM2_ESM.docx]

Supplementary Table 2. Bayesian information criterion (BIC)-based model selection for deciding the optimal number of PCs in the final model. Largest BIC value indicates best model.

A: BIC information for association mapping analysis for HG 2.5.7 SCN resistance

| Number of PCs | BIC | log Likelihood Function Value |
| --- | --- | --- |
| 0 | -2080.58 | -2071.76 |
| 1 | -2096.34 | -2069.14 |
| 2 | -2098.14 | -2069.88 |
| 3 | -2099.87 | -2069.97 |
| 4 | -2101.36 | -2070.73 |
| 5 | -2097.63 | -2069.57 |

B: BIC information for association mapping analysis for HG 1.2.3.5.6.7 SCN resistance

| Number of PCs | BIC | log Likelihood Function Value |
| --- | --- | --- |
| 0 | -206.59 | -207.75 |
| 1 | -209.65 | -209.18 |
| 2 | -212.31 | -210.36 |
| 3 | -212.87 | -210.76 |
| 4 | -210.34 | -209.79 |
| 5 | -209.82 | -209.23 |
