# Supplementary material for: Genome-wide association and genomic prediction identifies soybean cyst nematode resistance in common bean including a syntenic region to soybean Rhg1 locus
Source: Hortic Res. 2019 Jan 1;6:9. doi: 10.1038/s41438-018-0085-3 (PMC6312554; doi:10.1038/s41438-018-0085-3)
Supplement: Supplementary file 3 — Supplementary Table 3 Actual cyst count and predicted cyst count of HG type 2.5.7 by genomic prediction model on common bean accessions in the testing data set [file 41438_2018_85_MOESM3_ESM.docx]

Supplementary Table 3 Actual cyst count and predicted cyst count of HG type 2.5.7 by genomic prediction model on common bean accessions in the testing data set

| **PI** | **Actual**  **cyst count** | **Predicted cyst count** | **PI** | **Actual**  **cyst count** | **Predicted cyst count** |
| --- | --- | --- | --- | --- | --- |
| **PI313837** | 286 | 170 | **PI535395** | 215 | 90.7 |
| **PI313720** | 258 | 150.9 | **PI325687** | 206 | 81.2 |
| **PI313366** | 238 | 117.6 | **PI417679** | 205 | 226.4 |
| **G19833** | 227 | 185.9 | **PI533502** | 169 | 103.9 |
| **PI309825** | 226 | 155.1 | **PI399169** | 166 | 149.8 |
| **PI307788** | 211 | 215.3 | **PI417784** | 151 | 136.3 |
| **PI209486** | 207 | 172.9 | **PI449422** | 117 | 260.6 |
| **PI207203** | 200 | 166.4 | **PI319618** | 87 | 133.8 |
| **PI207420** | 163 | 163.3 | **PI319592** | 83 | 123.9 |
| **PI313727** | 162 | 114.8 | **PI319607** | 76 | 126.5 |
| **PI310883** | 142 | 154.2 | **PI417716** | 75 | 96.7 |
| **PI269210** | 122 | 155.6 | **PI533311** | 75 | 133.6 |
| **PI310556** | 112 | 152.9 | **PI417742** | 54 | 72.6 |
| **PI207182** | 110 | 139.9 | **PI325626** | 53 | 119.1 |
| **PI260418** | 110 | 167.1 | **PI451921** | 34 | 55.1 |
| **PI310726** | 106 | 120.9 | **PI318703** | 33 | 108.5 |
| **PI201387** | 82 | 91.5 | **PI417739** | 30 | 15.6 |
| **PI209498** | 64 | 62.7 | **PI326106** | 3 | 12.7 |
