# Supplementary material for: Genome-wide association and genomic prediction identifies soybean cyst nematode resistance in common bean including a syntenic region to soybean Rhg1 locus
Source: Hortic Res. 2019 Jan 1;6:9. doi: 10.1038/s41438-018-0085-3 (PMC6312554; doi:10.1038/s41438-018-0085-3)
Supplement: Supplementary file 4 — Supplementary Table 4 Actual cyst count and predicted cyst count of HG type 1.2.3.5.6.7 by genomic prediction model on common bean accessions in the testing data set [file 41438_2018_85_MOESM4_ESM.docx]

Supplementary Table 4 Actual cyst count and predicted cyst count of HG type 1.2.3.5.6.7 by genomic prediction model on common bean accessions in the testing data set

| **PI** | **Actual**  **cyst count** | **Predicted**  **cyst count** | **PI** | **Actual**  **cyst count** | **Predicted cyst count** |
| --- | --- | --- | --- | --- | --- |
| **PI415975** | 276 | 175 | **PI207148** | 782 | 128.3 |
| **PI313665** | 172 | 74.7 | **PI207279** | 405 | 73.3 |
| **PI312064** | 141 | 53.1 | **PI209486** | 393 | 109 |
| **PI313373** | 108 | 69 | **PI415886** | 171 | 106.4 |
| **PI310883** | 105 | 73.1 | **PI533528** | 168 | 79.8 |
| **PI207389** | 100 | 81.1 | **PI202835** | 118 | 70.4 |
| **PI309787** | 99 | 69.3 | **PI288016** | 115 | 73.2 |
| **PI207420** | 96 | 92.6 | **PI417634** | 108 | 61.7 |
| **PI310739** | 54 | 68.1 | **PI533373** | 88 | 87.7 |
| **PI511767** | 47 | 98 | **PI325722** | 80 | 151 |
| **PI313512** | 40 | 54.4 | **PI313532** | 49 | 71.8 |
| **PI533584** | 36 | 86 | **PI310561** | 47 | 72.2 |
| **PI308898** | 30 | 81 | **PI307806** | 40 | 81.1 |
| **PI198026** | 27 | 65 | **PI311942** | 35 | 52.7 |
| **PI325750** | 18 | 43.2 | **PI319595** | 27 | 111.7 |
| **PI165462** | 18 | 86.7 | **PI313495** | 16 | 47.1 |
| **PI165455** | 16 | 36 | **PI417784** | 6 | 57.1 |
| **PI313445** | 0 | 29 | **PI417778** | 2 | 55.1 |
