# Supplementary material for: Genome-wide association and genomic prediction identifies soybean cyst nematode resistance in common bean including a syntenic region to soybean Rhg1 locus
Source: Hortic Res. 2019 Jan 1;6:9. doi: 10.1038/s41438-018-0085-3 (PMC6312554; doi:10.1038/s41438-018-0085-3)
Supplement: Supplementary file 5 — Supplementary Table 5 Actual weight (gram) per 100 seeds and predicted weight (gram) per 100 seeds by genomic prediction model of the common bean accessions in the testing data set [file 41438_2018_85_MOESM5_ESM.docx]

Supplementary Table 5 Actual weight (gram) per 100 seeds and predicted weight (gram) per 100 seeds by genomic prediction model of the common bean accessions in the testing data set

| **PI** | **Actual weight** | **Predicted weight** | **PI** | **Actual weight** | **Predicted weight** |
| --- | --- | --- | --- | --- | --- |
| **PI309837** | 25 | 27.74 | **PI325684** | 2.8 | 8.49 |
| **PI309787** | 20 | 26.22 | **PI417667** | 20 | 29.77 |
| **PI310690** | 24.4 | 25.94 | **PI152208** | 60 | 55.75 |
| **PI165422** | 15 | 29.48 | **PI319587** | 32 | 34.16 |
| **PI430204** | 31 | 34.37 | **PI313470** | 32 | 33.34 |
| **PI207203** | 50.95 | 42.75 | **PI313583** | 22 | 23.34 |
| **PI313634** | 25 | 20.44 | **PI201296** | 54.4 | 31.53 |
| **PI293355** | 56.85 | 55.60 | **PI202835** | 25.2 | 23.91 |
| **PI313512** | 35 | 30.30 | **PI203920** | 27.9 | 31.90 |
| **PI313658** | 41 | 54.87 | **PI313254** | 19 | 46.80 |
| **PI299019** | 44.2 | 51.20 | **PI313809** | 40.53 | 32.07 |
| **PI310829** | 23 | 23.02 | **PI313837** | 68 | 56.23 |
| **PI309698** | 33.4 | 31.97 | **PI310586** | 23 | 25.27 |
| **PI241794** | 45 | 53.37 | **PI313630** | 35 | 30.75 |
| **PI325732** | 64.6 | 39.42 | **PI417790** | 35.4 | 31.37 |
| **PI309844** | 22.15 | 23.87 | **PI203936** | 26.9 | 31.34 |
| **PI309825** | 38.45 | 29.83 | **PI313782** | 16 | 22.64 |
| **PI313328** | 25 | 32.86 | **PI313537** | 61 | 41.35 |
