# Supplementary material for: Genome-wide association and genomic prediction identifies soybean cyst nematode resistance in common bean including a syntenic region to soybean Rhg1 locus
Source: Hortic Res. 2019 Jan 1;6:9. doi: 10.1038/s41438-018-0085-3 (PMC6312554; doi:10.1038/s41438-018-0085-3)
Supplement: Supplementary file 6 — Dear Editor [file 41438_2018_85_MOESM6_ESM.docx]

April 19, 2018

Dear Editor,

I am submitting the manuscript “Genome-wide association and genomic prediction identifies soybean cyst nematode resistance in common bean including a syntenic region to soybean *Rhg1* locus” for consideration of publication in Horticulture Research.

The manuscript used genome-wide association study to detect single nucleotide polymorphisms significantly associated with resistance to *Heterodera glycines* in the core collection of common bean, *Phaseolus vulgaris* and identified a syntenic region of SCN resistance in soybean.

All authors have agreed to submission. This work has not been published elsewhere. The authors have no conflict of interest.

Thank you for your consideration.

Glen L. Hartman
